# Association between 9p21 Genomic Markers and Ischemic Stroke Risk: Evidence Based on 21 Studies

**DOI:** 10.1371/journal.pone.0090255

**Published:** 2014-03-13

**Authors:** Xiaoqing Ni, Jiawei Zhang

**Affiliations:** 1 Department of Geriatrics, 107th Hospital of Chinese People’s Liberation Army, Binzhou Medical University, Yantai, Shandong, People’s Republic of China; 2 Department of Urology, 107th Hospital of Chinese People’s Liberation Army, Binzhou Medical University, Yantai, Shandong, People’s Republic of China; Mayo Clinic, United States of America

## Abstract

Epidemiological studies indicate a genetic contribution to ischemic stroke risk, but specific genetic variants remain unknown. Recently independent studies reported an association between coronary heart disease and single-nucleotide polymorphisms (SNPs) located at chromosome 9p21 (rs10757278 and proxy SNPs). Given that stroke is a common complication after myocardial infarction, several validation studies have been conducted among various ethnic populations to investigate if the same loci was associated with ischemic stroke (IS), but the results have been inconsistent. To investigate this inconsistency and derive a more precise estimation of the relationship, a meta-analysis of 34,128 cases and 153,428 controls from 21 studies was performed. Potential sources of heterogeneity including ethnicity, sample size, control source and ischemic stroke subtypes were also assessed. Overall, the summary odds ratio of IS was 1.11 (95% CI: 1.07–1.15, P<10^−5^) for rs10757278. In the subgroup analysis by ethnicity, significantly increased risks were found in East Asians (3188 cases and 4503 controls; OR = 1.14, 95% CI: 1.07–1.21, P<10^−5^) and Caucasians (30505 cases and 145153controls; OR = 1.08, 95% CI: 1.04–1.12, P<10^−5^) for the polymorphism; while no significant associations were found among African Americans (435 cases and 3772 controls; OR = 0.97, 95% CI: 0.63–1.51, P = 0.90) in all genetic models. In the subgroup analyses by IS subtypes, significant association was detected only in large vessel stroke group, while no significant associations among small vessel or cardioembolic stroke. When stratified by sample size, and control source, significantly increased risks were found for the polymorphism in all genetic models. This meta-analysis provides accurate and comprehensive estimates of the association of genetic variant at chromosome 9p21 and IS, but these associations vary in different ethnic populations.

## Introduction

Ischemic stroke (IS) is a leading cause of death and disability worldwide [1]. Traditional risk such as dyslipidemia, hypertension, atrial fibrillation smoking, and diabetes mellitus can only explain a small proportion of the observed clinical events [2]. However, a large proportion of the population attributable risk for ischemic stroke has remained unexplained [3]. Twin and familial aggregation studies suggest that the risk of stroke has a substantial genetic component [4], but the genes underlying this risk in the general population remain undetermined. Since the pathogenesis of ischemic stroke is yet to be elucidated completely, the candidate-gene approach is limited in power to detect novel disease-susceptibility genes.

Recently, significant advance was made in identifying susceptible genes underlying the risk of complex diseases such as type 2 diabetes and coronary disease through genome-wide association strategy (GWAS) [5]–[7]. The strongest association signal in the genome in GWAS for myocardial infarction (MI) and coronary artery disease (CAD) that has been published thus far comes from a number of SNPs with a high degree of linkage disequilibrium between each other on chromosome 9p21 [7]–[9]. Given the fact that ischemic stroke shares several common risk factors and pathophysiological mechanism with CAD and MI [10], [11], the genomic interval on chromosome 9p21 could be a candidate locus for IS as well. Only recently, several small studies have looked for an association between sequence variants on 9p21 and IS risk [8], [12], [13].

A number of studies have been conducted to investigate the association between chromosome 9p21 polymorphisms and the risk of IS in humans; however, these studies have yielded inconsistent result. Genetic association studies can be problematic to reproduce due to multiple hypothesis testing, relatively small sample size, population stratification, source of controls, publication bias, and phenotypic heterogeneity. In addition, with the increased studies in recent years among Asian, and other populations, there is a need to reconcile these data. We therefore performed a meta-analysis of the published studies to clarify this inconsistency and to establish a comprehensive picture of the relationship between genetic markers of chromosome 9p21 and IS.

## Materials and Methods

### Literature Search Strategy and Selection Criteria

Genetic association studies published before the end of August 2013 on ischemic stroke and polymorphisms within chromosome 9p21 gene were identified through a search of PubMed, ISI Web of Science, EMBASE and CNKI (Chinese National Knowledge Infrastructure) without language restrictions. Search term combinations were keywords relating to chromosome 9p21 (e.g., “chromosome 9p21”, “CDKN2A/B”, or “ANRIL”) in combination with words related to IS (e.g., “ischemic stroke”, “stroke”, “cerebral infarction”, “cerebral ischemia”, or “cerebrovascular disease”) and polymorphism or variation. We replaced one term each time until all possible combination mode were searched to avoid any missing literature. The titles and abstracts of potential articles were screened to determine their relevance, and any clearly irrelevant studies were excluded. The full texts of the remaining articles were read to determine whether they contained information on the topic of interest. All reference lists from the main reports and relevant reviews were hand searched for additional eligible studies.

Eligible studies had to meet all of the following criteria: (a) original papers containing independent data, (b) case–control or cohort studies, (c) identification of IS case was confirmed pathologically and (d) genotype distribution information or odds ratio (OR) with its 95% confidence interval (CI) and P-value. The major reasons for exclusion of studies were (a) overlapping data and (b) case-only studies, (c) family-based studies and review articles.

### Data Extraction

Information was carefully extracted from all eligible publications independently by two authors according to the inclusion criteria listed above. For each included study, the following data was extracted from each report according to a fixed protocol: first author, publication year, definition and numbers of cases and controls, diagnostic criterion, frequency of genotypes, source of controls, body mass index (BMI), age, sex, Hardy–Weinberg equilibrium (HWE) status, ethnicity and genotyping method. Discrepancies in data extraction were resolved by discussion between all authors through consensus. Studies with different ethnic groups were considered as individual studies for our analyses. Not all researchers use the same 9p21 SNPs, and most articles reported results for multiple SNPs (uniquely identified by their rs number). We extracted data for all SNPs used by the 21 included articles, but we report herein 1 common SNP (rs10757278) that was widely investigated, as other SNPs (rs2383207, rs2383206, rs10757274, and rs4977574) are in high linkage disequilibrium with rs10757278 (r^2^>0.85) [6], [8], [14], [15], [16].

### Statistical Methods

The strength of association between chromosome 9p21 polymorphisms and IS risk was assessed by OR with corresponding 95% CI. Deviation from Hardy–Weinberg equilibrium was examined by Chi-square test. If controls of studies were found not to be in HWE, sensitivity analyses were performed with and without these studies to test the robustness of the findings. The meta-analysis examined the association between chromosome 9p21 polymorphisms and the risk of IS: (1) allele contrast (effect of each additional risk allele), (2) dominant model (presence vs. absence of the risk allele), and (3) recessive model (presence vs. absence of two copies of the risk allele). Random-effects summary measure was calculated as inverse-variance-weighted average of the log odds ratio [17]. The results of random-effects summary were reported in the text because it takes into account the variation between studies. Heterogeneity was assessed with standard Q-statistic test and I^2^ test [18], [19].

In addition, sources of heterogeneity were investigated by stratified meta-analyses based on ethnicity, sample size (IS cases ≥500 or <500), ischemic stroke subtype and control source (hospital or population based study). Ethnic group was defined as Caucasian (i.e., people of white European origin), East Asian (e.g., Chinese, Japanese, Korean), and African American. BMI, sample size, age, sex and ethnicity were analyzed as covariates in meta-regression. The 95% CIs were constructed using Woolf’s method [20]. The significance of the overall OR was determined by the Z-test. Funnel plots and Egger’s linear regression test were used to assess evidence for potential publication bias [21]. In order to assess the stability of the result, sensitivity analyses were performed, each study in turn was removed from the total, and the remaining were reanalyzed. All the analyses were carried out with the STATA software version 10.0 (Stata Corporation, College Station, TX, USA). All P values are two-sided at the P = 0.05 level.

## Results

### Study Characteristics

**Figure 1:**
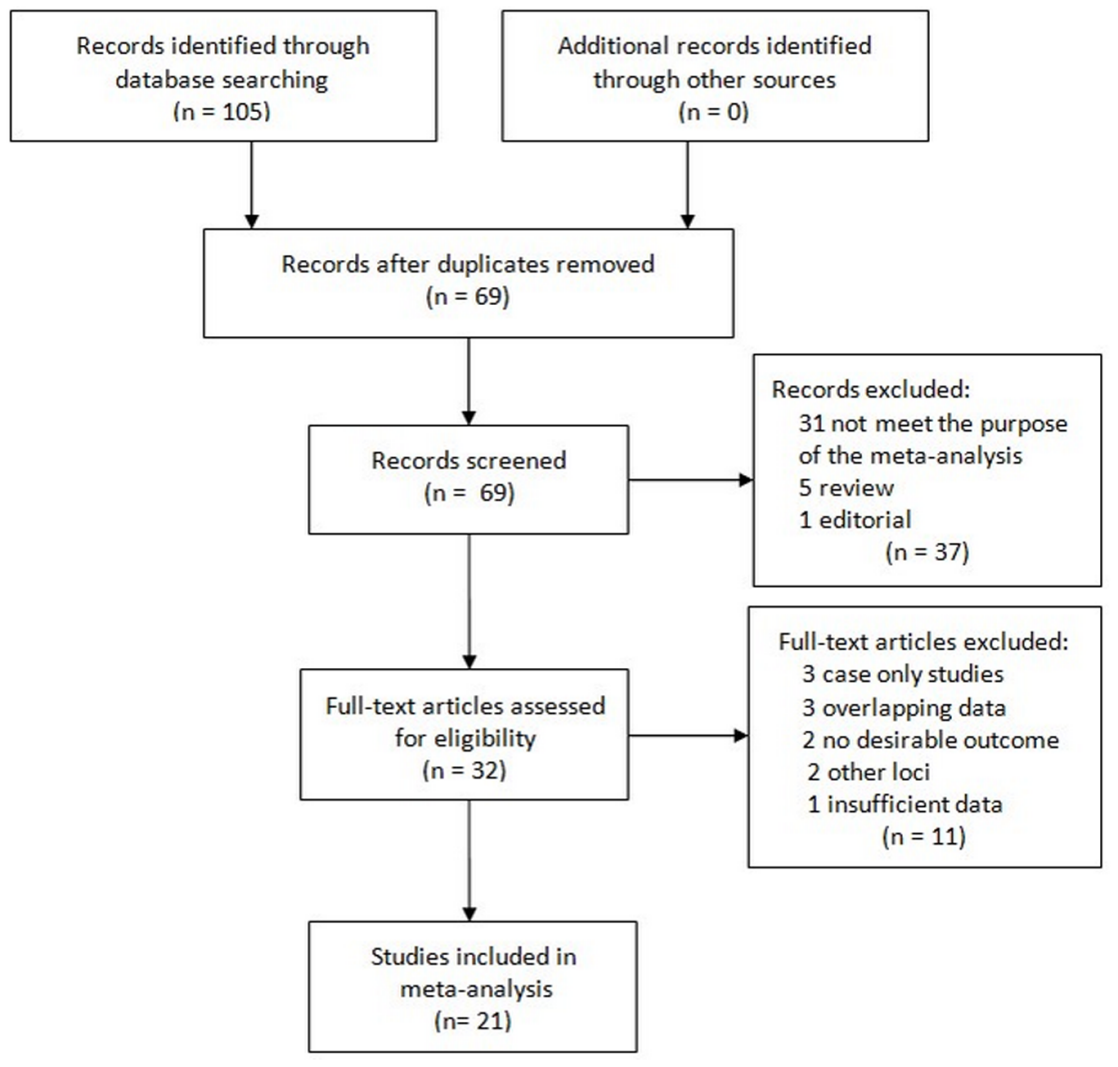
Study selection process.

The combined search yielded 105 references. 84 articles were excluded because they clearly did not meet the criteria or overlapping references (Figure 1). Finally, a total of 21 studies were finally included with 34,128 patients and 153, 428 controls [12]–[15], [22]–[38]. The detailed characteristics of the studies included in this meta-analysis are shown in Table 1. The polymorphism on chromosome 9p21 was found to occur in frequencies consistent with HWE in the control populations of the vast majority of the published studies. There are 26 data sets with 30505 cases and 145153 controls concerning Caucasians and 5 data sets with 3188 cases and 4503 controls concerning East Asians. For the African American, 4 data sets involved a total of 435 cases and 3772 controls.

**Table 1:** Characteristics of the studies included in the meta-analysis.

Reference	Year	Ethnicity	Ethnic groups	Polymorphism	Case	Match criteria for control	No. of cases/controls	Source of control	Genotyping method
Zee [12]	2007	American	Caucasian	rs10757274	CT or MRI confirmed	Age, ethnicity, smoking status	254/254	Population	NA
Matarin [13]	2008	American	Caucasian	rs2383207	IS per WHO criteria	Age, sex, ethnicity	249/268	Population	Chip
Helgadottir [14]	2008	European	Caucasian	rs10757278	CT or MRI confirmed	Ethnicity	705/14993	Population	Chip
Smith [15]	2009	Swedish	Caucasian	rs2383207	IS per WHO criteria	Age, sex, ethnicity, time of baseline investigation	2725/1840	Population	MassArray
Gschwendtner [22]	2009	European, African American	Caucasian, African American	rs10757278	CT or MRI confirmed	Age, sex, ethnicity, site of enrolment	932/4150	Population	TaqMan
Lemmens [23]	2009	Belgian	Caucasian	rs10757278	IS per WHO criteria	Sex, ethnicity	636/809	Population	Taqman
Karvanen [24]	2009	European	Caucasian	rs1333049	IS per ICD-9 criteria	Age, sex, ethnicity, site of enrolment	209/2064	Population	MassArray
Ikram [25]	2009	European	Caucasian	rs1537378	CT or MRI confirmed	Age, ethnicity	1164/18438	Population	Chip
Luke [26]	2009	Austrian	Caucasian	rs10757274	CT or MRI confirmed	Ethnicity	503/784	Population	Kinetic PCR
Yamagishi [27]	2009	American	Caucasian, African American	rs10757274	IS per ICD-9 criteria	Ethnicity	524/12856	Population	Taqman
Ding [28]	2009	Chinese	East Asian	rs10757278	IS per ICD-9 criteria	Ethnicity, resident area	999/1055	Population	TaqMan
Wahlstrand [29]	2009	Swedish	Caucasian	rs10757278	IS patients	Ethnicity, time of follow-up	163/5099	Hospital	MassArray
Hu [30]	2009	Chinese	East Asian	rs2383206	CT or MRI confirmed	Ethnicity	352/423	Hospital	SNPstream
Plant [31]	2011	American	Caucasian	rs10757278	CT or MRI confirmed	Age, sex, ethnicity	229/229	Population	Taqman
Olsson [32]	2011	Swedish	Caucasian	rs10757278	CT or MRI confirmed	Age, sex, ethnicity	834/665	Population	Golden Gate
Lin [33]	2011	Chinese	East Asian	rs1333049	IS per WHO criteria	Ethnicity	642/1361	Population	Taqman
Bellenguez [34]	2012	European, American	Caucasian	rs2383207	CT or MRI confirmed	Age, sex, ethnicity	1780/12253	Population	Chip
Traylor [35]	2012	European	Caucasian	rs2383207	CT or MRI confirmed	Ethnicity	12389/65004	Population	Chip, Taqman
Cheng [36]	2012	European, American	Caucasian	rs4977574	CT or MRI confirmed	Age, sex, ethnicity	6865/11395	Population	KASPar, Taqman
Zhang [37]	2012	Chinese	East Asian	rs10757278	IS per WHO criteria	Age, sex, resident area	1195/1664	Population	LDR
Heckman [38]	2013	American	Caucasian, African American	rs2383207	IS per WHO criteria	Ethnicity	879/824	Population	MassArray

### Meta-analysis Results

**Figure 2:**
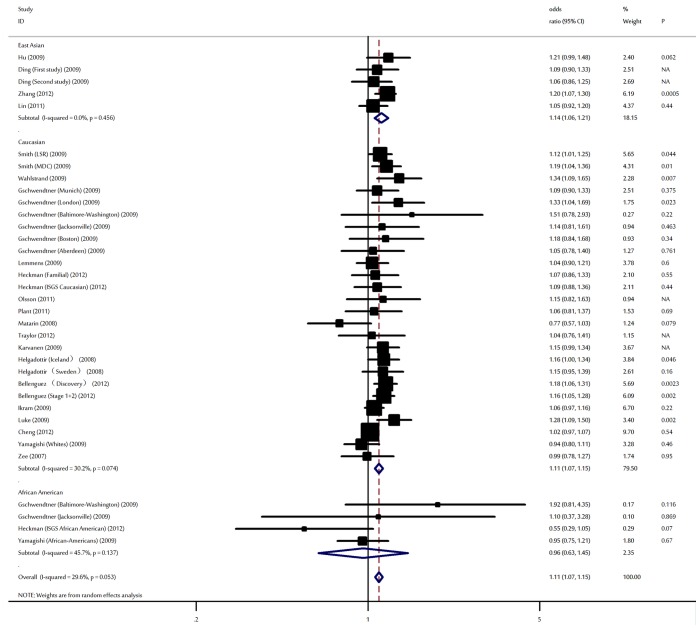
Meta-analysis of studies on the association of rs10757278 on chromosome 9p21 with ischemic stroke.

The main results of this meta-analysis were listed in Table 2. In the overall analysis, the risk allele of rs10757278 polymorphism was significantly associated with elevated IS risk. Under a random-effect model, the per-allele OR for IS was 1.11 (95% CI: 1.07–1.15, P<10^−5^; Figure 2) with corresponding results under dominant and receive genetic model of 1.19 (95% CI: 1.11–1.25, P<10^−5^) and 1.23 (95% CI: 1.19–1.29, P<10^−5^), respectively.

**Table 2:** Meta-analysis of the chromosome 9p21 genetic marker on ischemic stroke risk.

Sub-group analysis	No. of data sets	No. of case/control	Allele contrast					Dominant model					Recessive model				
			OR (95% CI)	P-value	P(Q)^a^	I^2^ (%)	P(Q)^b^	OR (95% CI)	P-value	P(Q)^a^	I^2^ (%)	P(Q)^b^	OR (95% CI)	P-value	P(Q)^a^	I^2^ (%)	P(Q)^b^
Overall	35	34128/153428	1.11 (1.07–1.15)	<10^−5^	0.05	30		1.19 (1.11–1.25)	<10^−5^	0.19	21		1.23 (1.19–1.29)	<10^−5^	0.07	33	
Ethnicity							0.14					0.20					0.11
Caucasian	26	30505/145153	1.11 (1.07–1.15)	<10^−5^	0.07	30		1.18 (1.14–1.27)	<10^−5^	0.24	15		1.26 (1.19–1.31)	<10^−5^	0.19	14	
East Asian	5	3188/4503	1.14 (1.06–1.21)	<10^−5^	0.46	0		1.19 (1.10–1.31)	<10^−5^	0.39	0		1.17 (1.05–1.32)	<10^−4^	0.36	7	
African American	4	435/3772	0.96 (0.63–1.45)	0.83	0.14	46		1.06 (0.90–1.26)	0.58	0.14	10		1.08 (0.98–1.22)	0.13	0.46	0	
Sample size							0.03					0.05					0.18
Small	23	5340/42445	1.11 (1.05–1.17)	0.001	0.12	22		1.16 (1.10–1.24)	<10^−5^	0.20			1.20 (1.05–1.39)	<10^−5^	0.17	12	
large	12	28788/110983	1.10 (1.06–1.15)	<10^−5^	0.09	27		1.21 (1.09–1.24)	<10^−5^	0.31	8		1.25 (1.17–1.29)	<10^−5^	0.48	0	
Control source							0.02					0.13					0.08
Hospital	2	515/5522	1.27 (1.10–1.47)	0.001	0.49	0		1.28 (1.15–1.46)	<10^−4^	0.27	16		1.45 (1.18–1.99)	0.0008	0.10	21	
Population	33	33613/147906	1.10 (1.06–1.14)	<10^−5^	0.08	26		1.18 (1.10–1.25)	<10^−5^	0.12	11		1.22 (1.17–1.31)	<10^−5^	0.03	30	
IS subtypes							<10^−5^					<10^−5^					<10^−5^
Large vessel	9	6226/89235	1.15 (1.10–1.19)	<10^−5^	0.54	0		1.19 (1.08–1.30)	<10^−5^	0.27	19		1.24 (1.07–1.45)	<10^−4^	0.39	0	
Cardioembolic	5	4744/78485	1.03 (0.95–1.13)	0.47	0.09	50		1.17 (0.95–1.46)	0.31	0.05	62		1.22 (0.92–1.68)	0.46	0.13	20	
Small vessel	6	4272/80149	1.02 (0.98–1.07)	0.31	0.87	0		1.01 (0.96–1.06)	0.17	0.58	0		1.07 (0.98–1.19)	0.08	0.46	0	
Other determined causes	2	535/15657	1.01 (0.85–1.19)	0.91	0.33	0		1.09 (0.95–1.25)	0.26	0.12	7		1.52 (0.53–4.35)	0.41	0.27	0	
Undetermined causes	2	3358/15657	1.02 (0.96–1.08)	0.46	0.62	0		1.07 (0.97–1.26)	0.71	0.48	0		1.10 (0.97–1.26)	0.21	0.54	0	

a Cochran’s chi-square Q statistic test used to assess the heterogeneity in subgroups.

b Cochran’s chi-square Q statistic test used to assess the heterogeneity between subgroups.

Allele contrast (effect of each additional risk allele).

Dominant model (presence vs. absence of the risk allele).

Recessive model (presence vs. absence of two copies of the risk allele).

**Figure 3:**
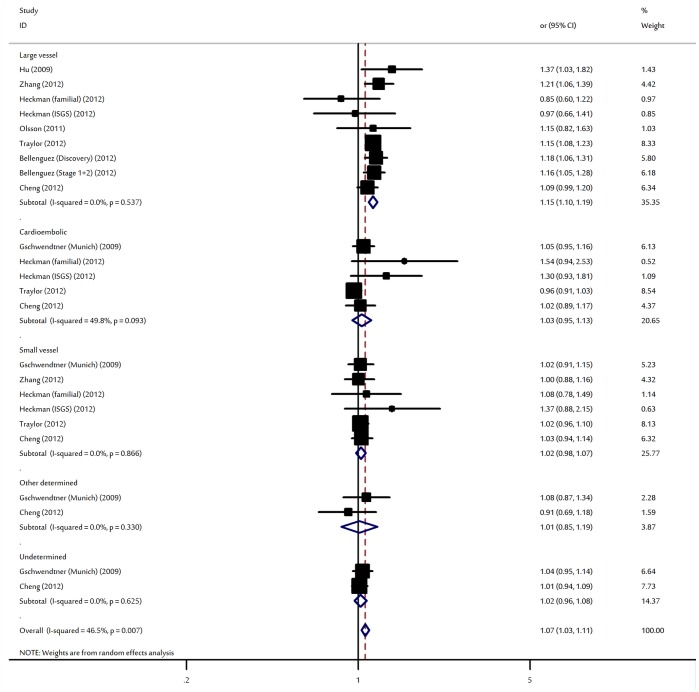
Forest plot for the association between rs10757278 and ischemic stroke risk by stroke subtype status.

When studies were stratified for ethnicity, significant risks were found among East Asians in all genetic model [allele contrast: OR = 1.14, 95% CI: 1.06–1.21; dominant model: OR = 1.19, 95% CI: 1.10–1.31; recessive model: OR = 1.17, 95% CI: 1.05–1.32]. Similar results were also found in Caucasian populations [allele contrast: OR = 1.11, 95% CI: 1.07–1.15; dominant model: OR = 1.18, 95% CI: 1.14–1.27; recessive model: OR = 1.26, 95% CI: 1.19–1.31]. However, no significant association was found for African American populations in all genetic models. Subsidiary analyses of sample size yielded a per-allele OR for small studies of 1.11 (95% CI: 1.05–1.17, P = 0.001) and large studies of 1.10 (95% CI: 1.06–1.15, P<10^−5^). By considering control source subgroups, the OR was 1.10 (95% CI: 1.06–1.14, P<10^−5^) in population-based controls compared to 1.27 (95% CI: 1.10–1.47, P = 0.001) in hospital-based controls. In the subgroup analyses by ischemic stroke subtype, significant associations were found for large vessel stroke in all genetic modes [allele contrast: OR = 1.15, 95% CI: 1.10–1.19; dominant model: OR = 1.19, 95% CI: 1.08–1.30; recessive model: OR = 1.24, 95% CI: 1.07–1.45]. However, we failed to detect any association between small vessel stroke, cardioembolic stroke, or other type of stroke risk and the polymorphism (Figure 3 and Table S1). After adjusting for multiple testing using Bonferroni correction, all significant associations for rs10757278 under the three different genetic models remained.

Although the formal test for heterogeneity was not significant (P>0.05), we conducted meta-regression as there were also grounds for considering the ethnicity, sample size, IS subtype, and clinical characteristics of cases and controls (age, BMI, and sex distribution) as potential sources of heterogeneity. However, the meta-regression showed that none of these covariates significantly contributed to the heterogeneity among the individual study results except for ethnicity (P = 0.01) and IS subtype (P = 0.002).

### Sensitivity Analyses and Publication Bias

Sensitivity analysis indicated that no single study influenced the pooled OR qualitatively, suggesting that the results of this meta-analysis are stable (Figure S1). The shape of the funnel plots was symmetrical (Figure S2). The statistical results still did not show publication bias in these studies (Begg test, P = 0.55; Egger test, P = 0.45).

## Discussion

Genome-wide association studies have identified a locus for risk of coronary artery disease on chromosome 9p21 [7]–[9]. Recent studies have also analyzed the association between 9p21 and overall ischemic stroke, with diverse outcomes [22]–[27]. The present meta-analysis provides the most comprehensive assessment of the risk of IS and 9p21 variant (rs10757278). Its strength was based on the accumulation of published data giving greater information to detect significant differences. In total, the meta-analysis involved 21 studies for IS which provided 34,128 cases and 153, 428 controls.

Our results demonstrated that the rs10757278 polymorphism on chromosome 9p21 is a risk factor for developing ischemic stroke. In the stratified analysis by ethnicity, significant associations were found in East Asian and Caucasian populations for the polymorphism in all genetic models. However, no significant associations were detected among African populations. There are several possible reasons for such differences. In fact, the frequencies of the risk-association alleles in chromosome 9p21 are similar in European and East Asian populations, but substantially lower in African descent [22], [27], [37], [38]. Thus, failing to identify any significant association in African populations could be due to substantially lower statistical power caused by the relatively lower prevalence of the risk allele. In addition, study design or small sample size or some environmental factors may affect the results. Most of these studies did not consider most of the important environmental factors. It is possible that variation at this locus has modest effects on IS, but environmental factors may predominate in the progress of IS, and mask the effects of this variation. Specific environmental factors like lifestyle and diabetes that have been already well studied in recent decades. The unconsidered factors mixed together may cover the role of the polymorphism. Furthermore, different populations usually have different linkage disequilibrium patterns. A polymorphism may be in close linkage with another nearby causal variant in one ethnic population but not in another. The rs10757278 polymorphism may be in close linkage with different nearby causal variants in different populations. Nevertheless, owing to the limited number of relevant studies among African Americans included in this meta-analysis, the observed ethnic difference in this meta-analysis is also likely to be caused by chance because studies with small sample sizes may have insufficient statistical power to detect a slight effect or may have generated a fluctuated risk estimate. Thus, further studies including a wider spectrum of subjects to investigate the role of chromosome 9p21 variants in this population will be needed.

Meta-analysis is often dominated by a few large studies, which markedly reduces the evidence from smaller studies. However, in the stratified analysis according to sample size, significantly increased IS susceptibility in risk allele carriers rs10757278 polymorphism was also found both in large and small studies for all genetic models.

Ischemic stroke itself has a number of subtypes with the most common being large-vessel atherosclerotic stroke, small-vessel disease, and cardioembolism. As ischemic stroke subtypes was the main source of heterogeneity in our meta-analysis, we performed subgroup analyses by IS subtypes. We found that the risk allele has an increased risk in large-vessel stroke subgroup but not in small-vessel or cardioembolic stroke subgroup. This finding is in line with previous family history studies on ischemic stroke subtypes, showing a greater risk associated with large vessel stroke than small vessel stroke [39]. Recently, Zhang et al. [37] reported that family history of stroke further increased the stroke risk to 2.37-fold in subjects carrying 4 copies of G-allele of rs10757274 and rs10757278, and also increased the risk of stroke recurrence (2.45-fold). Thus, a combination of the risk variants on 9p21.3 with family stroke history could help to predict an individual’s risk of stroke. The reason for the observed stroke-specific difference in the risk conferred by the rs10757278 polymorphism is unknown. It has been suggested that genetic predisposition may differ for these subtypes [40], and of note, most monogenic forms of stroke predispose to individual stroke subtypes [40]. This genetic heterogeneity seems likely to reflect heterogeneity in the underlying pathogenic mechanisms and reinforces the need for the consideration of stroke subtypes separately in research and clinical contexts.

The association between ischemic stroke and SNPs at a locus previously associated with coronary artery disease and diabetes suggest that ischemic stroke shares common pathophysiological pathways with these diseases. Recently, a common variant near the CDKN2B gene in the chromosome 9p21 locus is associated with a lower ankle-brachial index which is a simple and reliable method to detect peripheral arterial disease [41]. The cardiovascular disease-associated regions at the chromosome 9p21 locus are adjacent to the last exons of a long noncoding RNA (lncRNA), ANRIL (also known as CDKN2BAS) [42]. Two cyclin-dependent kinases inhibitors, CDKN2A and CDKN2B (encoding p15INK4B, p16INK4A, and p14ARF) lie close to the association spot (∼100 kb from the associated SNPs). CDKN2A/B are known to be repressed by Polycomb proteins during cell growth and then activated during senescence [43]. There is strong evidence to support the role of ANRIL in the regulation of the CDKN2A/B locus through histone modification [44], [45]. ANRIL has been proposed to regulate senescence at the CDKN2A locus, and it showed a senescence-dependant role in proliferation [44]. These findings emphasize the importance of ANRIL in cell proliferation and regulation of the locus CDKN2A/B in a cell line directly involved in the pathogenesis of atherosclerosis.

In summary, this study provides the most comprehensive evidence that 9p21 is a susceptibility locus in ischemic stroke, particularly in East Asian and Caucasian populations. More important, these variants may have different degrees of influence on various subtypes of ischemic stroke. Larger studies of different ethnic populations, especially strict selection of patients, well-matched controls, are needed to confirm our findings. An improved understanding of the pathogenesis of IS will be beneficial in the diagnosis of prodromal symptoms and in establishing appropriate therapeutic intervention to prevent the onset and the progression of IS.

## Supporting Information

Figure S1 **Result of sensitivity analyses for rs10757278 polymorphism and ischemic stroke risk.**
(TIF)

Figure S2 **Funnel plot for rs10757278 polymorphism and ischemic stroke risk.**
(TIF)

Table S1 Per-allele OR for rs10757278 variant and risk of IS subtype stratified by ethnic groups.
(DOCX)

Checklist S1 **CONSORT Checklist.**
(DOC)
