# Supplementary figures and images for: Association between 9p21 Genomic Markers and Ischemic Stroke Risk: Evidence Based on 21 Studies
Source: PLoS One. 2014 Mar 13;9(3):e90255. doi: 10.1371/journal.pone.0090255 (PMC3953076; doi:10.1371/journal.pone.0090255)

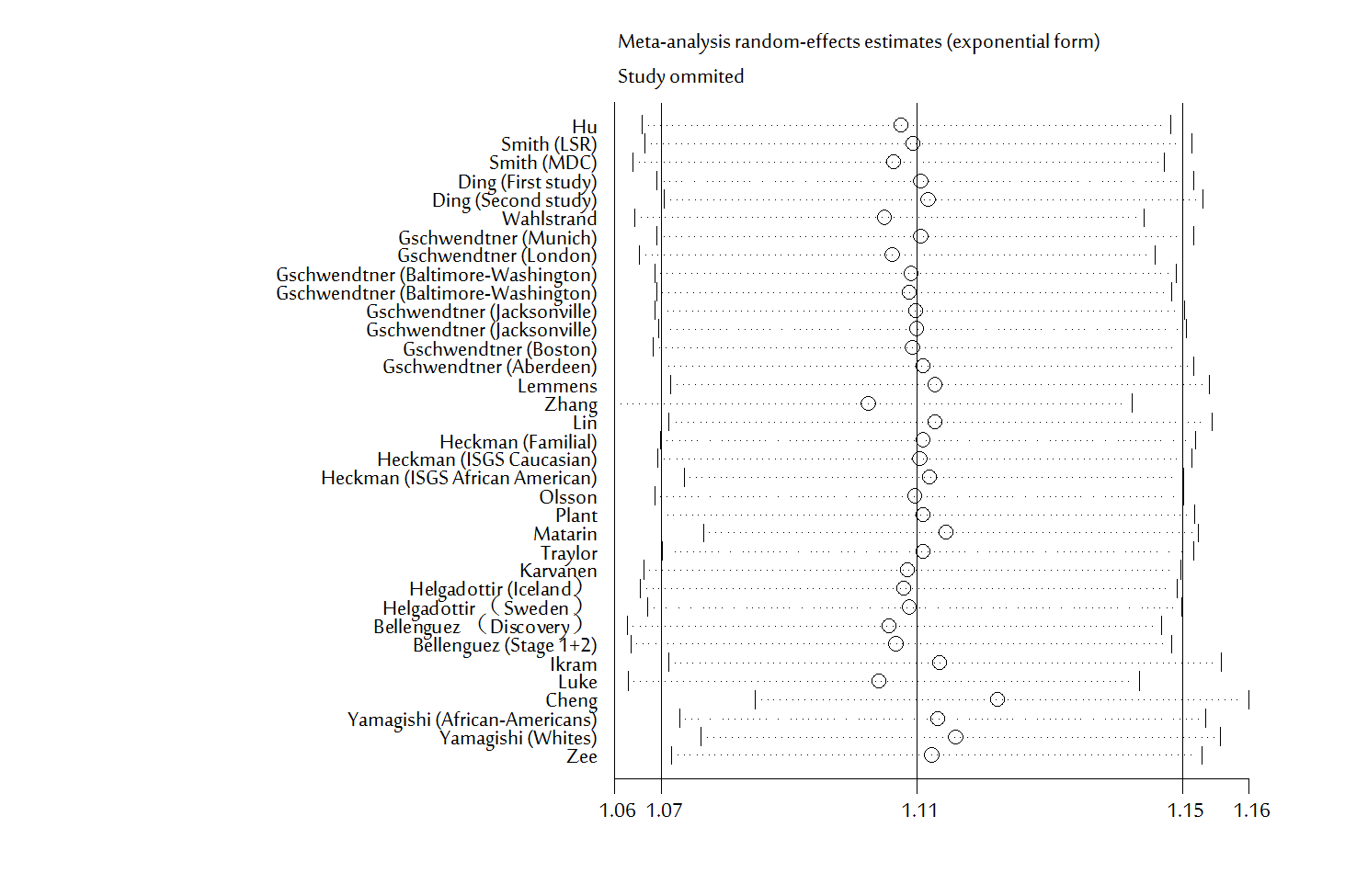

Supplement: Figure S1 — Result of sensitivity analyses for rs10757278 polymorphism and ischemic stroke risk. (TIF) [file pone.0090255.s001.tif]

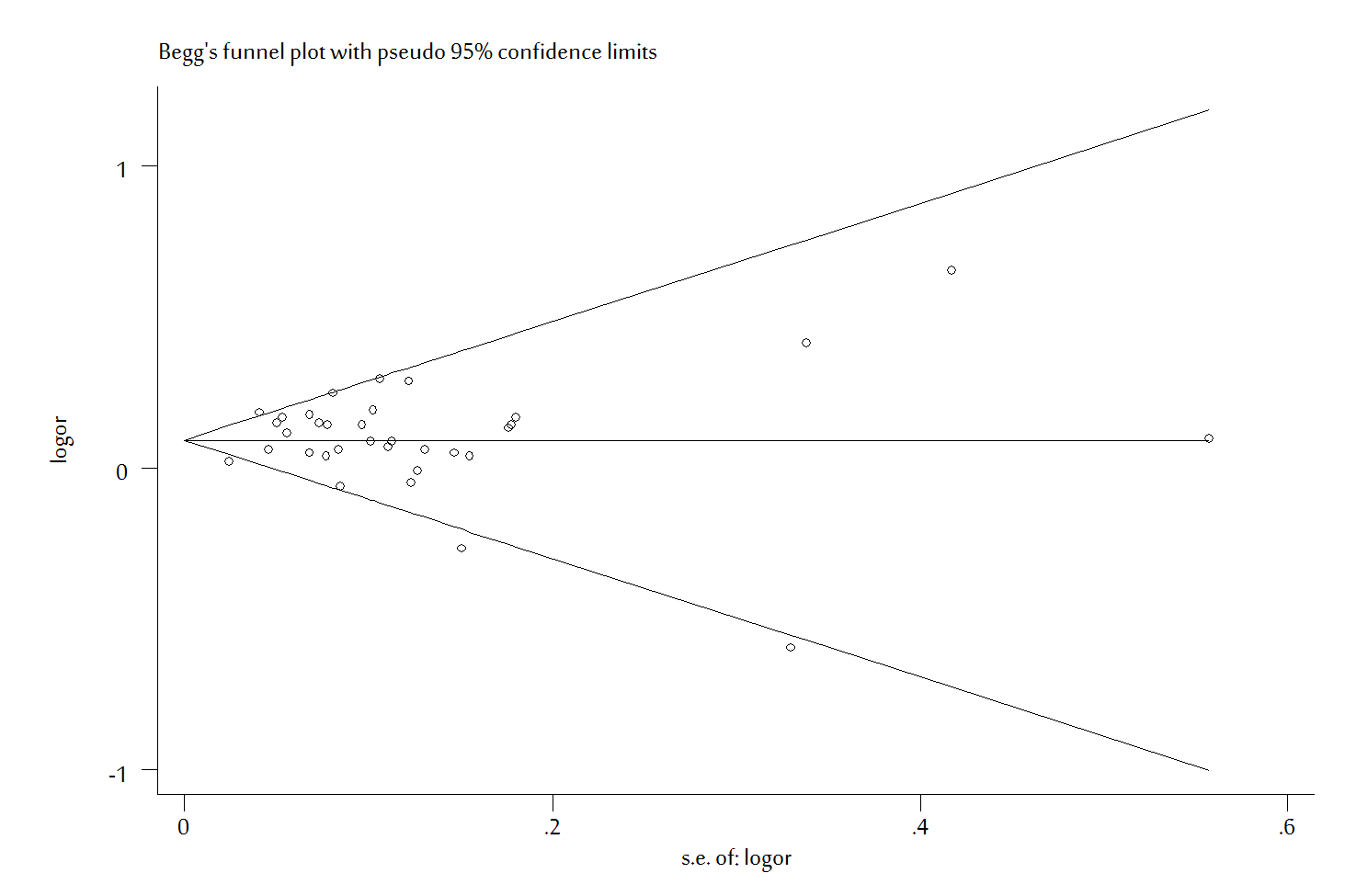

Supplement: Figure S2 — Funnel plot for rs10757278 polymorphism and ischemic stroke risk. (TIF) [file pone.0090255.s002.tif]
