# Supplementary material for: Association between 9p21 Genomic Markers and Ischemic Stroke Risk: Evidence Based on 21 Studies
Source: PLoS One. 2014 Mar 13;9(3):e90255. doi: 10.1371/journal.pone.0090255 (PMC3953076; doi:10.1371/journal.pone.0090255)
Supplement: Table S1 — Per-allele OR for rs10757278 variant and risk of IS subtype stratified by ethnic groups. (DOCX) [file pone.0090255.s003.docx]

| Ethnic group | IS subtype | No. of datasets | No. of cases/controls | OR (95%CI) | P-value |
| --- | --- | --- | --- | --- | --- |
| Caucasian | Large vessel | 7 | 5350/87025 | 1.14 (1.09-1.19) | <10^-5^ |
|  | Cardioembolic | 5 | 4715/78369 | 1.03 (0.95-1.13) | 0.47 |
|  | Small vessel | 5 | 3762/78369 | 1.03 (0.98-1.08) | 0.28 |
|  | Other determined causes | 2 | 535/125657 | 1.01 (0.85-1.19) | 0.91 |
|  | Undetermined causes | 2 | 3358/125657 | 1.02 (0.96-1.08) | 0.46 |
| East Asian | Large vessel | 2 | 846/2094 | 1.24 (1.10-1.40) | 0.001 |
|  | Small vessel | 1 | 466/1664 | 1.00 (0.87-1.15) | 0.99 |

**Table S1** Per-allele OR for rs10757278 variant and risk of IS subtype stratified by ethnic groups.
